# The emerging pathogen *Enterocytozoon hepatopenaei* drives a degenerative cyclic pattern in the hepatopancreas microbiome of the shrimp (*Penaeus vannamei)*

**DOI:** 10.1038/s41598-022-19127-2

**Published:** 2022-08-30

**Authors:** Jesús Antonio López-Carvallo, Roberto Cruz-Flores, Arun K. Dhar

**Affiliations:** 1 Centro de Investigación Científica y de Educación Superior de Ensenada (CICESE), Carretera Ensenada-Tijuana No. 3918, Zona Playitas, 22860 Ensenada, Baja CA Mexico; 2 Aquaculture Pathology Laboratory, School of Animal and Comparative Biomedical Sciences, The University of Arizona, 1117 Lowell Street, Tucson, AZ 85721 USA

**Keywords:** Microbiology, Bacteria, Fungi, Pathogens

## Abstract

The microsporidian *Enterocytozoon hepatopenaei* (EHP) is an emerging pathogen that causes substantial economic losses in shrimp (*Penaeus* spp.) aquaculture worldwide. To prevent diseases in shrimp, the manipulation of the gut microbiota has been suggested. However, prior knowledge of the host-microbiome is necessary. We assessed the modulation of the microbiome (bacteria/fungi) and its predicted functions over the course of disease progression in shrimp experimentally challenged with EHP for 30 days using high throughput 16S rRNA and ITS amplicon sequencing. Infection grade was assessed for the first time by quantitative digital histopathology. According to the infection intensity, three disease-stages (early/developmental/late) were registered. During the early-stage, EHP was not consistently detected, and a high diversity of potentially beneficial microorganisms related to nutrient assimilation were found. In the development-stage, most of the shrimp start to register a high infection intensity related to a decrease in beneficial microorganisms and an increase in opportunistic/pathogenic fungi. During late-stage, animals displayed different infection intensities, showed a displacement of beneficial microorganisms by opportunistic/pathogenic bacteria and fungi related to pathogen infection processes and depletion of energetic reserves. The degenerative cyclic pattern of EHP infection and its effects on beneficial microorganisms and beneficial functions of the shrimp hepatopancreas microbiome are discussed.

## Introduction

The white leg shrimp, *Penaeus vannamei,* is an economically important species that represents 52.9% of the total production of crustacean aquaculture (9.4 million tons) worldwide^1^. Even though production has been steadily increasing in recent years, shrimp aquaculture is in constant challenge for the emergence and spread of new diseases such as hepatopancreatic microsporidiosis (HPM) caused by an emergent pathogen *Enterocytozoon hepatopenaei* (EHP). This disease was first reported in 2001, affecting black tiger shrimp *P. monodon* and was tentatively termed monodon slow growth syndrome (MSGS)^2^. In 2009, EHP was formally described from *P. monodon*^3^ postlarvae/juveniles and the disease was later referred as HPM^4^.

Hepatopancreatic microsporidiosis has now been reported in Thailand, Vietnam, India, China, Indonesia, Malaysia, Venezuela and has kept spreading to other countries where shrimp culture takes place^5^. Presently, EHP is considered one of the major threats to shrimp culture^6^ and is imposing major limitations to shrimp farming across Asia^5,7^. Since sanitary measures around the world have historically failed to contain the spread of pathogens in shrimp farming^8^ and only a few functional therapeutic methods are available to treat crustacean diseases^9^, researchers have focused their efforts on exploring novel alternatives to prevent diseases in shrimp including the modulation of the host-microbiome^10,11^. The last is a promising alternative as the microbiome is closely related to particular functions of the host^11–13^ and can be used as a driver for success in shrimp farming^10^, which may help to prevent/control EHP infection.

The modulation of the microbiome has been used to improve shrimp health, growth performance and resistance to disease which in turn favors higher farm production^14^. This modulation can be attained at a farm level in shrimp culture by using probiotics, prebiotics, synbiotics, and by creating an environment favorable to probiotics (e.g. biofloc)^14,15^. However, to modulate the microbiome in a beneficial manner that promotes increased growth requires prior knowledge of health and diseased shrimp microbiomes^10^.

The microbiome status of an organism can be deciphered by using new generation technologies such as high throughput sequencing through the amplification and sequencing of the hypervariable regions of genes such as 16S rRNA for bacteria and internal transcribed spacer (ITS) for fungi^16^. Amplicon sequencing by high throughput methods allows the quantification of microorganisms’ taxa abundance, assess microbial diversity and predicts the microbiome functions based on the prevalent microorganisms’ taxa^17^. These methods have been used by many researchers to obtain a high-resolution map of the host-microbiome^10^. Furthermore, they allow a better insight into the complex interactions between host and microorganisms when the health condition are affected by the presence of pathogens^10^.

Insights on the modulation of the hepatopancreatic microbiota (bacteria and fungi) and associated functions by EHP in *P. vannamei* are greatly lacking. This study evaluated the composition, diversity and functions of hepatopancreas microbiota of the pacific white shrimp *P. vannamei* infected with EHP for 30 days following a pathogen challenge. The findings greatly enhanced our understanding of disease stage-specific community assembly patterns in the shrimp hepatopancreas micro-ecosystem upon infection with EHP.

## Methods

### Shrimp acquisition

Specific pathogen-free (SPF) shrimp, *P. vannamei* (~ 5 g) originating in the Oceanic Institute (Oahu, Hawaii, USA) were used for the experimental bioassay conducted in the Aquaculture Pathology Laboratory of the University of Arizona. All animals used in this study are certified SPF and these stocks have consistently tested negative for the OIE-listed and the OIE non-listed pathogens including EHP. Before the experiment shrimp were acclimatized for 2 weeks in a 100 L recirculating culture system (25 °C) provided with gentle aeration and fed with a commercial shrimp diet (Rangen, 40% protein).

### Experimental challenge

Experimental challenge was conducted following a cohabitation challenge method. Briefly, upon acclimatization, animals were tagged by cutting a small piece of the uropod to identify challenged SPF shrimp from the EHP-infected population. Before SPF *P. vannamei* were exposed to EHP-infected shrimp (day 0), a total of two SPF shrimp were sampled and hepatopancreas tissue was aseptically excised, one part was flash-frozen in liquid nitrogen and the other section was fixed in Davison alcohol-formalin-acetic acid (AFA) solution. On the following day, a total of 36 SPF shrimp were transferred to an experimental tank that held known EHP-infected *P. vannamei* shrimp. The cohabitation challenge was conducted for 30 days while experimental animals were sampled throughout the challenge period. Transmission of EHP by co-habitation was preferred as it is a fast and cost-effective method for parasite propagation that closely simulates natural routes of EHP infection in a shrimp farm^18,19^.

During the experimental challenge, the organisms were fed daily a commercially pellet shrimp diet (Rangen, 40% protein) for the duration of the study, and temperature and salinity in the tank were held to 25 °C and 33 parts per unit, respectively. Through the entire duration of the challenge experiment, on every third day two (third and sixth day) to three (from the ninth day onwards) tagged SPF shrimp were sampled. The hepatopancreas was excided into two equal parts of tissue, one part was flash-frozen and kept at − 80 °C for microbiome analysis, and the other one was fixed in Davidson AFA solution for in situ hybridization analysis. The hepatopancreas tissue was selected as a target tissue since EHP develops and replicates in this tissue^3^. This study was performed following standard protocols of the University of Arizona-Aquaculture Pathology Laboratory which is an USDA and ISO (17043 and 170225) accredited laboratory for Crustacean Disease Diagnosis.

### Quantitative digital histopathology using in situ hybridization

As a strategy to evaluate the infection grade of the cohabitation challenged shrimp, a quantitative digital histopathology analysis was performed. In situ hybridization (ISH) was performed following methodologies previously reported to detect EHP^20^ where EHP-specific primers 510 F (5′-GCCTGA GAG ATG GCT CCC ACG T-3′) and 510 R (5′-GCG TAC TAT CCC CAG AGC CCG A-3′) are used and tailed with digoxigenin-11-dUTP (Integrated DNA Technologies^®^, San Diego, CA, USA) at 3′ end. The severity of EHP infection was graded from G0–G4 following a semi-quantitative grading scheme^21^. Briefly, Grade 0 indicates no signs of infection, Grade 1 indicates signs of infection by the pathogen but at levels that may be below to those needed for clinical disease, Grade 2 moderate signs of infection shown by number and severity of pathogen caused lesions, Grade 3 moderate to high signs of disease shown by number and severity of pathogen caused lesions and Grade 4 high numbers of pathogen caused lesions and tissue destruction.

The ISH glass slides that contained the dissected tissue area were scanned and digitalized using a MoticEasyScan Pro 6 Pathology Slide Scanner (Meyer Instruments, Inc., TX, USA). Digital images were processed with Image-Pro Plus 10.0 (Media Cybernetics, Bethesda, MD, USA) according to López-Carvallo et al.^22^. In each slide, three random images at 10× magnification were selected considering the infected areas as representative of the infection of that particular shrimp. This information was used to calculate the EHP infection index (EII) using the following formula: EII (%) = ((CAE/TCA) × 100), where CAE is the coverage area of EHP (µm^2^), and TCA is the total coverage area of the analyzed region of the affected tissue (µm^2^). A range of EII percentages was assigned to the severity of the infection grade scale proposed by Lightner^21^. The EII percentages were normalized by arcsin, and a one-way analysis of variance was performed to assess significant differences as a function of infection grade and stage of the disease.

### Bacterial and fungal DNA isolation

A total of 30 flash-frozen samples of hepatopancreas from tagged SPF shrimp that were kept in cohabitation with EHP infected shrimp were used to extract bacterial and fungal genomic DNA (gDNA) from each animal. The total gDNA was extracted from the sampled tissue (20 mg) using the Genomic DNA Isolation Kit (Norgen Biotek Corp^®^, ON, Canada) and the Maxwell^®^ 16 Cell LEV DNA Purification Kit (Promega, WI, USA) according to the manufacturer’s instructions.

The integrity of total gDNA was corroborated by gel electrophoresis, and quality and concentration were measured by a NanoDrop 2000/2000c™ (Thermo Fisher Scientific, Waltham, MA, USA). The isolated DNA were sent for NGS at OmegaBioservices, Norcross, GA. PCR reactions were performed using 16S rRNA primers IlluminaF (5′-CCTACGGGNGGCWGCAG-3′) and IlluminaR (5′-ACTACHVGGGTATC-TAATCC-3′) while the internal transcribed spacer (ITS) was amplified using the primers ITS1F (5′-C TTG GTC ATT TAG AGG AAG TAA-3′) and ITS4R (5′-T CCT CCG CTT ATT GAT ATG C-3′).

### 16S rRNA V3-V4 and ITS amplicons sequencing

Before construction of the libraries, gDNA quality was assessed using TapeStation (Agilent^®^). A total of 30 libraries for bacteria analysis and 30 libraries for fungal analysis were prepared using the kit KAPA HyperPlus (Roche^®^, Basile, Switzerland) according to the manufacturer’s recommendations. Library quality was analyzed with Agilent Bioanalyzer 2100 system (Agilent^®^, Santa Clara, CA, USA) and sequenced with Illumina Mi-Seq^®^ platform (San Diego, CA, USA) on mode pair-end (2X300). The generation of libraries and sequencing was performed by OmegaBioservices (Norcross, GA, USA). A total of 1,331,020 16S rRNA V3–V4 and 883,460 ITS raw reads were generated via sequencing.

### Bioinformatics and statistical analysis

To increase the number of runs using different parameters to evaluate the microbiome of *P. vannamei* infected with EHP, we decided to use a graphical user interface tool called EasyMAP^17^. This tool integrates Quantitative Insights Into Microbial Ecology 2 (QIIME2, 2018.4.0 version)^23^, Linear Discriminant Analysis Effect Size (LefSe, 1.0.8.post1 version)^24^, and Phylogenetic Investigation of Communities by Reconstruction of Unobserved States (PICRUSt, 1.1.3 version)^25^ pipelines, and incorporates the database of Greengenes, SILVA and UNITE^17^. Databases generated from high through sequencing for bacterial and fungal analysis were analyzed separately using the same pipeline except for the database. Greengenes Database was used to identify the bacterial taxa and UNITE to identify the fungus taxa. The raw reads were first analyzed with the FastQC software^26^ to assess the quality. The pair-end libraries containing raw read, sample metadata and manifest files were uploaded at the EasyMAP portal (http://easymap.cgm.ntu.edu.tw/). These files are used to identify libraries and define the statistical group for each sample for later analysis^17^.

For all statistical analyses, we used three groups according to the disease stage classification (early, development and late), where individual organisms were considered as biological replicates. This decision was made according to multiple clustering analyses since it has been confirmed that organisms clustered in the same group are related to similar physiological conditions, tissue or environment^27,28^. Pearson’s correlation and PCoA mainly sustain the clustering by disease-stage compared to infection grade and time of exposure with the pathogen.

Since sequence files were already demultiplexed, the manifest file was used according to the EasyMAP platform^17^. QIIME2 plug-in q2-dada2 was used to trimmed low-quality read (Q < 25), merge pair-end high-quality reads and remove chimeric sequences. No chimeric sequences were used for operational taxonomic unit (OTU) assignment. The taxonomic analysis (OUT table construction, taxonomy assignment, relative abundances tables by taxa) was assessed using the QIIME2 plug-in q2-feature-classifier. A 97% of sequence similarity was considered for sequence clustering and similar sequences from the same taxa were clustered into one OTU. The phylogenetic tree generation was performed by the plug-in q2-alignment and q2-phylogeny. Frequency bars graphs for phyla and Heatmaps for genera visualization were plotted in R.

The taxonomy differential abundance analysis was performed by the LEfSe plug-in using the KEGG database at level three and Wilcoxon test (α < 0.05). Taxonomy differential abundance was only registered when LDA > 2. Taxa abundance that were differentially expressed was plotted using bar, heatmap and circular cladogram graphics provided by EasyMAP platform or plotted using R.

The platform EasyMAP provided statistical results for alpha and beta diversity. Observed OTUs, Shannon diversity index (H) and Simpson’s diversity index (D) were used as standard statistical tools of alpha diversity and Principal Coordinates Analysis (PCoA) for beta diversity analysis. Alpha diversity was assessed by Kruskal–Wallis of all groups and pair-wise; (*P* < 0.05) and visualized using box plots. The beta diversity was assessed by PERMANOVA (*P* < 0.05) using Jaccard methodology (measures presence/absence) and PCoA was used to visualize microorganism composition and abundance similarity/dissimilarity between samples.

Microbiome function was predicted by using taxa abundance differentially expressed and the PICRUSt plug-in which mapped Greengenes IDs to the corresponding KEGG pathway. This tool has been used to predict the bacterial functional composition in crustaceans’ guts^29^. The predicted microbiome function was plotted by circular graphics using a log10 LDA score.

**Figure 1:**
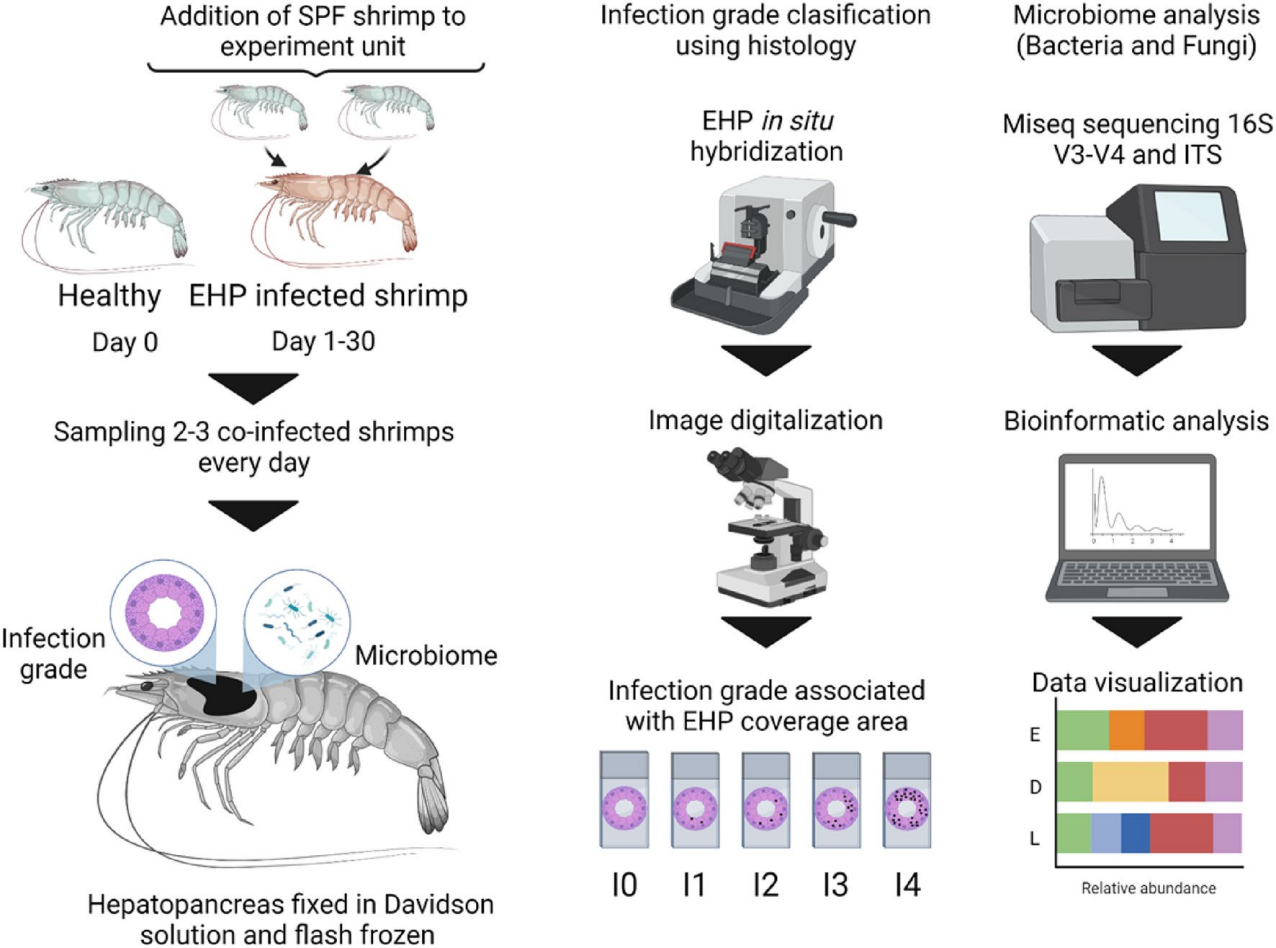
Work diagram and sampling collection. Experimental design to assess the bacterial and fungi community structure and predict hepatopancreatic microbiome functions in shrimp co-infected by EHP for 30 days. This image was created with BioRender (http://biorender.com/).

The heatmaps, bar, and circular graphs, and hierarchical clustering (Pearson correlation) were performed by using ggplot2 and hclus, respectively, using R software in Rstudio^30^. Relative abundance shown in heatmaps figures were log2 transformed to visualize taxa with low relative abundance (< 1%). The general experimental design can be visualized as a graphical abstract to aid readers’ comprehension (Fig. 1). For all replicates, a descriptive ID was added including sample day (D0–D30), infection grade (I0–I4), and the replicate number (R1–R3) to facilitate tracking of the microbiome changes caused by EHP infection (e.g. D0_I0_R1). In addition, the statistical groups (disease stages) were marked in three different colors (Early: green; Development: red; Late: blue) for bacteria and fungi analyses. Particularly in fungi, analysis of differentially expressed and microbiome function prediction was performed by using subgroups where early-stage was divided in control, D1N, D2N, D2I; development-stage: D3E D3L, D4E and D4L; late-stage: D5E and D5L. Control corresponds to organisms sampled on day 3; D1 on day 6; D2 on day 9 and 12; D3 on day 15 and 18; D4 on day 21 and 24; D5 on day 27 and 30. The N was used to describe non-infected organisms, I for infected organisms at the early disease stage, E for organisms with infection grades of I1 and I2, and L for organisms with infection grades of I3 and I4. These sub-groups were assessed in fungi taxa due to the variability pattern among samples.

## Results

### Digital histopathology: detection of EHP and quantification of the severity of infection

**Figure 2:**
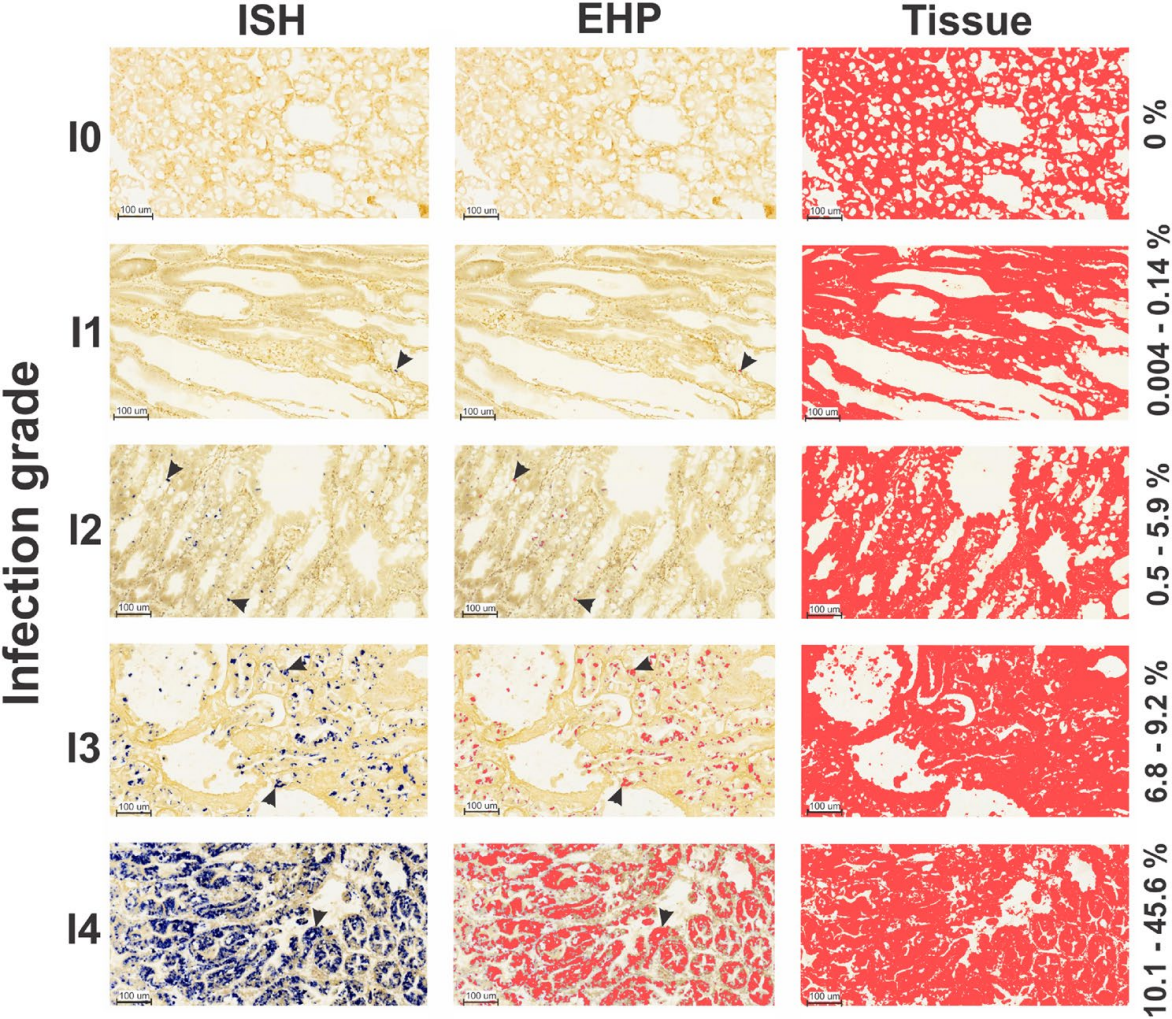
EHP infection intensity grade scale. Infection grade was assessed by in situ hybridization (ISH) using the EHP infection index (EII) and assigned by the methodology proposed by Lightner^21^. Each infection grade category (I0–I4) was represented for the first time by a value range of EII (%) shown at the right side of the image. The figure shows how the EHP and tissue area are separately quantified using ×10 image to calculate the EII according to each infection stage proposed by Lightner^21^. Black arrows mark EHP detection.

**Figure 3:**
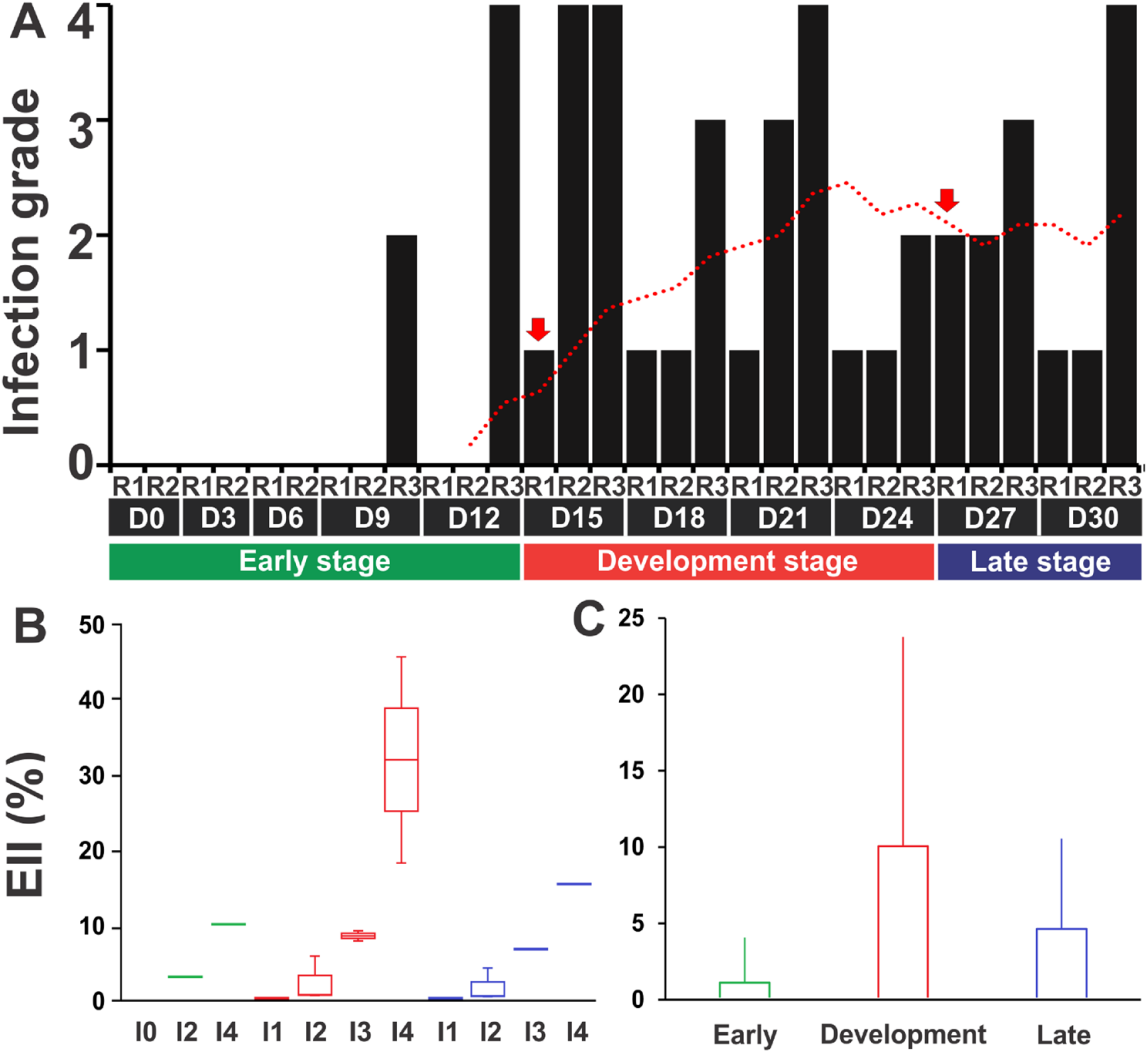
Evaluation of EHP infection. (**A**) EHP infection grade (I0–I4) registered during the bioassay and EHP infection index (EII) values according to (**B**) infection grade by each disease stage and (**C**) mean value for each disease stage. Black bars denote the infection grade of each organism sampled. The red dotted line represents the average of black bars considering 3 sampling days. Red arrows represent the beginning and the end of each disease stage. Color bars cluster the organisms from early (green), development (red), or late (blue) stages of the disease. R: replicate (sampled shrimp); D: day (sampling day). EII values are represented as mean ± standard deviation.

Five infections grades in the hepatopancreas of co-infected shrimp were assigned according to Lightner^21^: I0, I1, I2, I3 and I4 which were related (*P* < 0.05) to an EII value of 0%, 0.004–0.14%, 0.51–5.92%, 6.85–9.26% and 10.15–45.67% respectively (Fig. 2). A total of three stages of the disease were used to describe the infection cycle: early, development and late (Fig. 3A) which was supported by the EII assessed by infection grade (I0–I4) by each disease stage (early-late) (Fig. 3B) and mean value for each disease stage (Fig. 3C).

Due to the variability of the infection process of EHP, it was not possible to ensure replicates for each infection grade in each disease stage. For this reason, the statistics of microsporidiosis development were descriptive. The early-stage consisted of the first 12 post-infection days (dpi) where the lower EII mean value (0.92 ± 3.06%) was registered. EHP was only detected in one sampled shrimp on 9 and 12 dpi. After the early-stage, all sampled shrimp were positive to EHP (I1–I4). The development-stage started at 15 and ended at 24 dpi and was accompanied by the higher mean value of the EII (8.08 ± 13.75%). This stage was characterized by the abrupt increase of infected shrimp at high-infection grades (I3 and I4) and shrimp at I4 registered the highest EII values (31.97 ± 19.37%) throughout the bioassay. In the late-stage (at 27 to 30 dpi), the number of shrimp with a high infection intensity (I3 and I4) was reduced and the EII mean value decreased (5.44 ± 6.28%). At the late-stage, we observed what appears to be an asymptotic pattern where shrimp are in a cyclic infection process. According to these results, EHP proliferates possibly until shrimp reach infection grade I4 and then parasite load starts decreasing until shrimp reach infection grade I1. This pattern could be repeated throughout the culture cycle.

### Microbiome composition analysis

For bacteria and fungi taxonomic assignments, a total of 532,841 and 658,492 non-chimeric sequence reads (Q > 25) were used for OTUs table construction using the Greengenes and UNITE database, respectively. A total of 1895 OTUs for bacteria and 213 OTUs for fungi were found and used as a reference for clustering libraries reads into OTUs. Among bacteria, the assigned taxonomic hierarchy classification allowed identifying two domains, bacteria (97–100%) and archaea (< 3%). A total of 20 bacteria (11 unclassified) and 3 archaea phyla were identified and classified further into 234 (215 unclassified) genera. Among fungi, one domain was identified, fungi. A total of 3 fungi phyla were identified and classified further into 32 (5 unclassified) genera.

**Figure 4:**
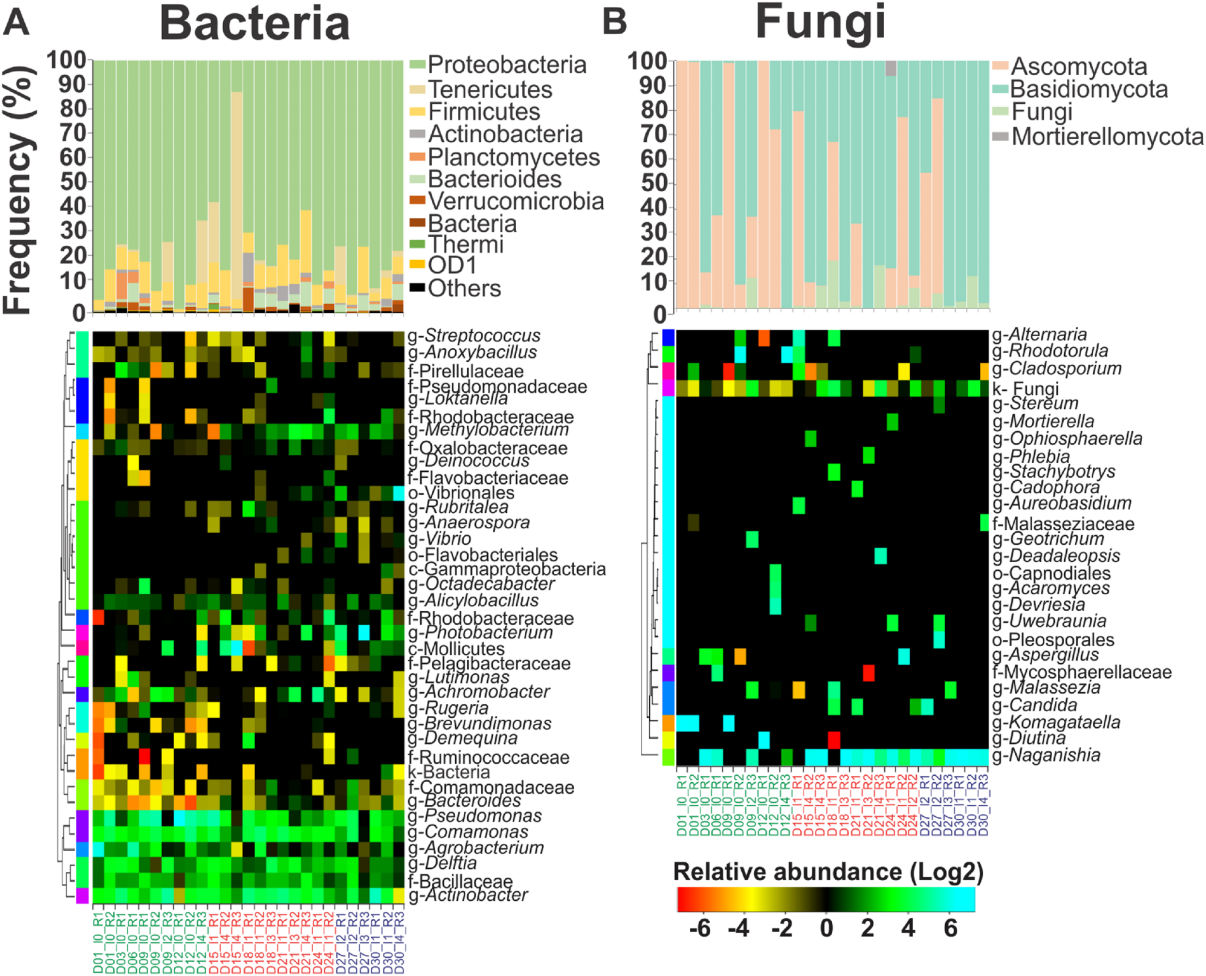
Shrimp microbiome at phyla and genera level registered at different disease stages. Microbiome modulation for (**A**) bacteria and (**B**) fungi taxa. Relative abundance is expressed as a percentage for phyla, where only phyla with higher relative abundance than 1% in at least one of the replicates are shown. Heatmaps using Log2 transformed relative abundance were used to cluster (y axis) genera present in similar conditions. Only genera with higher relative abundance than 1% in at least one of the replicates are shown. Shrimp were grouped (x axis) by the disease stage (early in green, development in red and late in blue) for easy visualization of the microbiome change between healthy and infected shrimp. The heatmaps color key ranges from orange for low abundant (< 1%) microorganisms to cyan for high abundant microorganisms (1–90%). Letters p- (phyla), c- (class), o- (order), f- (family) and g- (genera) on the left side of the taxa name represent the group level.

The most representative phyla found among bacteria included Proteobacteria (12–98%), Firmicutes (1–24%) and Tenericutes (0.3–82%) (Fig. 4A) while Basidiomycota (0–99%) and Ascomycota (0–99%) were most abundant among fungi (Fig. 4B). Differences among disease stages at a phylum level were only seen in fungi domain, where a reduction of Ascomycota phyla was accompanied by an increase of the Basidiomycota phyla at the late-stage of the disease.

The most represented genera in bacteria were *Pseudomonas* (0.7–80%) (Fig. 4A) and in fungi *Naganishia* (0–97%) (Fig. 4B). In bacteria, *Pseudomonas*, *Comamonas*, *Agrobacterium*, *Delftia* and *Actinobacteria* were clustered separately from other genera such *Streptococcus*, *Anoxybacillus*, *Loktanella*, *Methylobacterium* and *Vibrio* (Fig. 4A). In fungi, most of the genera were clustered together except for *Naganisha* (Fig. 4B). This clustering allowed the identification of microorganisms that may be found in a particular microenvironment (e.g. physicochemical parameters and physiological stages of the host).

### Microbiome modulation in shrimp hepatopancreas infected by EHP

**Figure 5:**
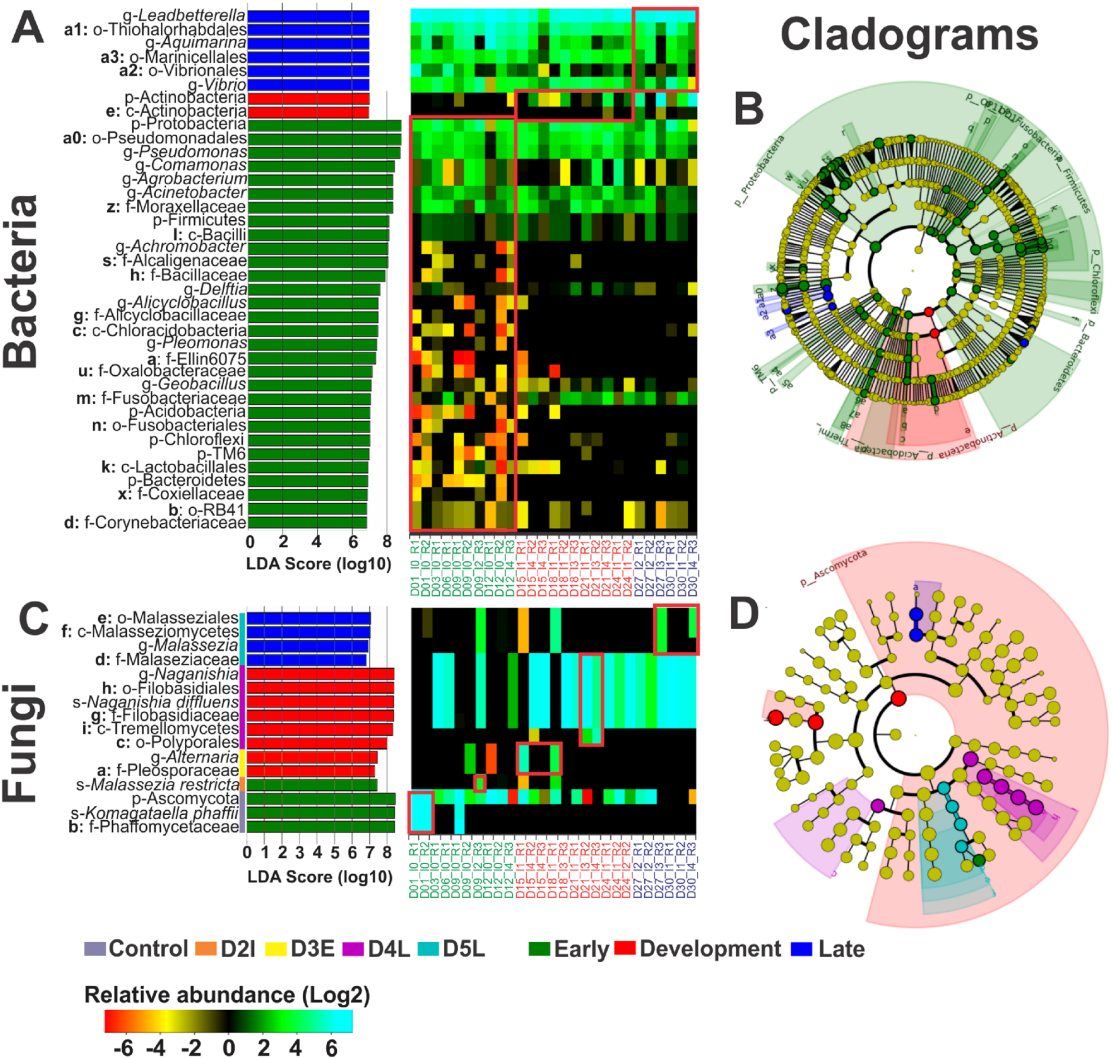
LEfSe results for enriched taxa of shrimp at early, development a late stage of the disease. Differentially expressed taxa visualized together with heatmap and cladogram graph for bacteria (**A**,**B**) and fungi (**C**,**D**), respectively. Differentially expressed taxa shown as log10 LDA score for each disease classification. Heatmaps using Log2 transformed relative abundance were used to cluster (x axis) genera by disease stage (early in green, development in red and late in blue). Red squares highlight the differentially expressed taxa in each disease stage condition. Cladograms represent the phylogenetic relation between microorganisms detected at the different disease stages and are marked with a bold alphabetical letter to locate the name of the taxa in the log10 LDA score graph. The heatmaps color key ranges from orange for low abundant (< 1%) microorganisms to cyan for high abundant microorganisms (1–90%). The top 30 overrepresented taxa are shown. Letters p- (phyla), c- (class), o- (order), f- (family) and g- (genera) on the left side of the taxa name represent the group level. For fungi, a subgroup of disease-stage was used: control and D2I from the early-stage, D3E and D4L for the development-stage and D5L for the late-stage. For fungi, subgroups of disease-stage were used: control, D1N, D2N, D2I for early-stage, D3E D3L, D4E and D4L for development-stage and D5E and D5L for late-stage.

According to the taxonomic differentially expressed analysis in bacteria (*P* < 0.05), a higher number of differentially expressed taxa were found in early-stage (59 taxa) compared to the development and late-stage (2 and 6 taxa, respectively). The genera *Pseudomonas*, *Comamonas* and *Agrobacterium* were highly related to the early-stage of the disease (*P* < 0.05), the class Actinobacteria to the development-stage (*P* < 0.05), and the genera *Leadbetterella*, *Aquimarina* and *Vibrio* to the late-stage of the disease (*P* < 0.05) (Fig. 5A). Differentially expressed bacteria (*P* < 0.05) at the different disease stages were phylogenetically different between disease stages (Fig. 5B). In the fungi domain, the genus *Komagataella* was related to the early-stage (*P* < 0.05), the genera *Naganisha* and *Alternaria* to the development-stage (*P* < 0.05) while the genus *Malassezia* was related to the late-stage (*P* < 0.05) (Fig. 5C). The fungi at different disease stages were not grouped by phylogenetic analysis (Fig. 5D). Some bacteria and fungi genera can be found in different disease-stages, but their abundance is associated to the disease stage.

### Bacterial richness and diversity

**Figure 6:**
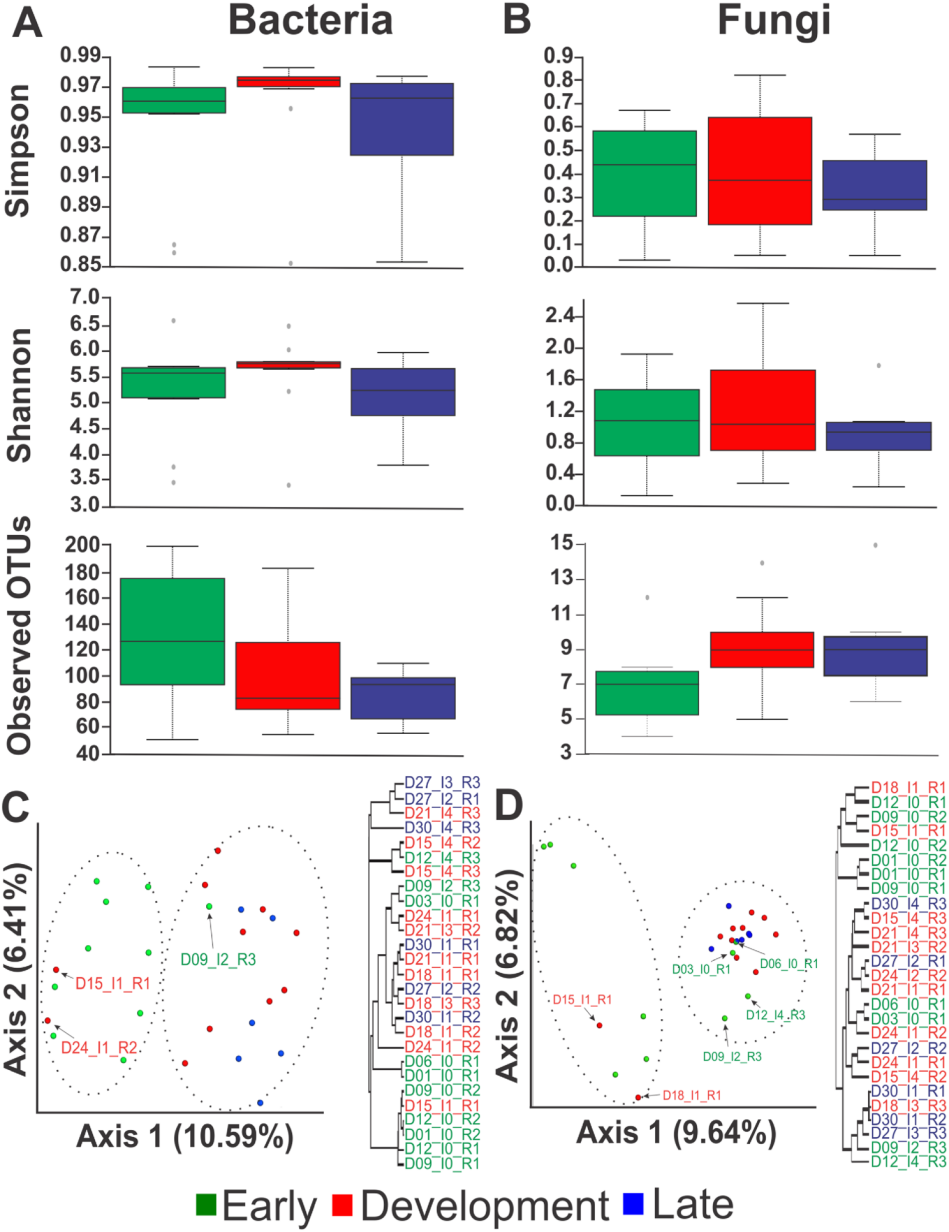
Box plots of three different alpha indexes and PCoA plot of beta index. Alpha indexes were assessed by Simpson and Shannon index and observed OTUs for (**A**) bacteria and (**B**) fungi. Beta index visualized with a PCoA and hierarchical clustering of Pearson’s correlation for (**C**) bacteria and (**D**) fungi at different disease stages. The colors green (early), red (development) and blue (late) represent the groups of organisms at different disease stages.

Alpha diversity was assessed by Simpson index and Shannon index for diversity and Observed OTUs for richness. For bacteria, the diversity indexes in the shrimp hepatopancreas were high in all disease-stages (Fig. 6A), while in fungi the diversity indexes were low (Fig. 6B). Non-statistical differences were found between disease stage in bacteria and fungi using Simpson index (*P* = 0.15, *P* = 0.77), Shannon index (*P* = 0.12, *P* = 0.71) and Observed OTUs (*P* = 0.17, *P* = 0.09), respectively. However, in bacteria, a higher diversity was registered at the early-stage and a lower score at the late-stage. In contrast, in fungi, we observed an inverse pattern compared to bacteria.

In both bacteria and fungi, the beta diversity plotted in PCoA graphics separated organisms into two different groups (*P* < 0.05). Shrimp in the early-stage of the disease were separated from shrimp at the development and late-stage. The same aggrupation was visualized in clustering replicate by Pearson's correlation (Fig. 6C,D). In some cases, organisms from the development-stage with the lowest infection grade were grouped with early-stage organisms. While some organisms from the early-stage with high infection grades (I2 and I4) were grouped with organisms at the development-stage (Fig. 6C,D).

### Identification of microbiome functionality

**Figure 7:**
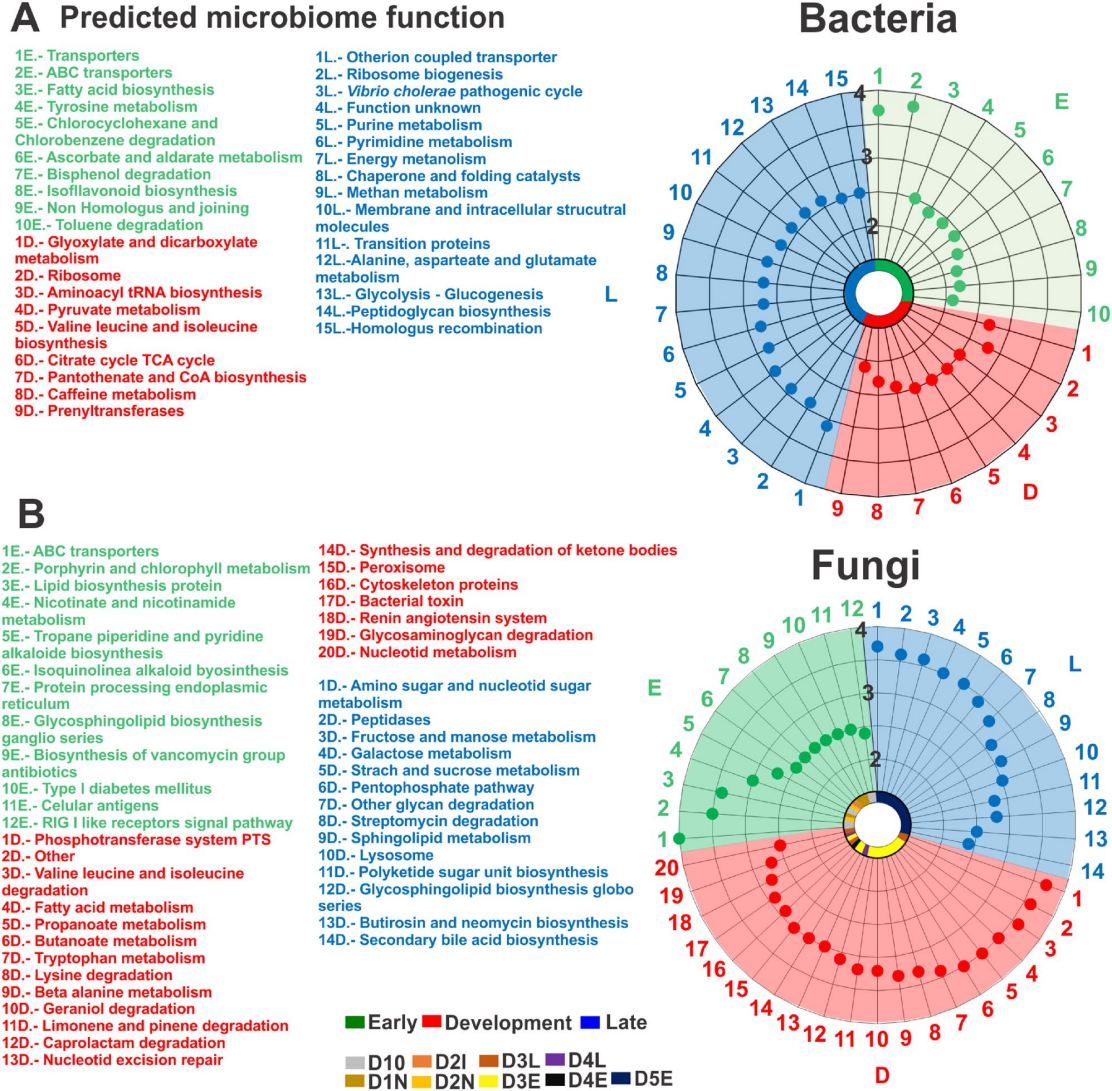
Difference of enriched KEGG metabolic pathways between disease stages groups. The graph shows the predicted microbiome functions according to (**A**) bacteria and (**B**) fungi found at the early (E; green), development (D; red) and late (L; blue) stages of the disease. Circular graphs show log10 LDA score (1–4) of the enriched KEGG metabolic pathways. For fungi, subgroups of disease-stage were used: control, D1N, D2N, D2I for early-stage, D3E, D3L, D4E and D4L for development-stage and D5E and D5L for late-stage. This image was created using EasyMAP^17^ and KEGG database^64^.

The integration of LEfSe and PICRUSt results showed significant change (*P* < 0.05) in the microbiome functionality among disease-stages. In general, we observed a transition from a healthy hepatopancreatic microbiome flora to a pathobiome. The microbiome function of bacteria at the early-stage of the disease (*P* < 0.05) were mainly associated with membrane transporter, fatty acid biosynthesis and other metabolic pathways associated to nutrient acquisition (Fig. 7A). This was highly similar to the predicted microbiome functions of fungi at the early-stage (ABC transporters, lipid biosynthesis protein, among others) (Fig. 7B).

During the development-stage, although the microbiome of shrimp was still partially related to nutritional function, some pathways related to the bacteria transcription (e.g. ribosome and aminoacyl tRNA biosynthesis) processes in the bacteria group (Fig. 7A) and bacterial toxin production in the fungi group were registered (*P* < 0.05) (Fig. 7B).

At the late-stage, most of the enriched metabolic pathways (*P* < 0.05) in bacteria were different from those found in fungi. Bacterial microbiome predicted function (*P* < 0.05) was mainly related to opportunistic or pathogenic bacteria proliferation and bacterial infection process (e.g. *Vibrio cholerae* pathogenic cycle and peptidoglycan biosynthesis) (Fig. 7A). The fungi microbiome predicted function were mainly related to protein and carbohydrates degradation (e.g. amino sugar and nucleotide sugar metabolism, peptidases and other glycan degradation) (Fig. 7B).

## Discussion

Hepatopancreatic microsporidiosis (HPM) is an emergent threat to sustainable shrimp farming worldwide. Our understanding of host–pathogen interactions in HPM will allow the development of strategies to mitigate risk of disease outbreaks in shrimp farming. Our results show that infection by EHP in *P. vannamei* can be detected as early as 9 dpi when using the co-habitation challenge model coupled with in situ hybridization (ISH). The earlier detection of EHP in this study compared to other authors, that report EHP detection 14 dpi^18^, could be attributed to the differences in many exogenous (e.g. parasite load, water quality) and endogenous (e.g. physiological state) factor modulating the parasite’s infection process in shrimp^19,31^. In any case, the capability of detecting EHP in less than 10 dpi could have positive diagnostic implication since combining ISH and real-time PCR could be an effective means in detecting the parasite early in a culture cycle.

Our data revealed that EHP pathogenesis in challenged shrimp shows a well-defined pattern. During the first 12 dpi (early-stage), the presence of EHP was undetectable except for one organism at each day 9 and 12 dpi. This was followed by a development-stage where the pathogen proliferated in the hepatopancreatic tissue. Thereafter, shrimp entered the late-stage of disease progression which appears to have a cyclic infection pattern, where organisms from infection grade I4 might decrease to I1, and repeated the cycle, without reaching I0 again. To our knowledge, this is the first time that a cyclic pattern of EHP proliferation has been proposed in *P. vannamei*. Cyclic infection patterns have been previously related to chronic infections in mammals and in other invertebrates^32,33^. The interaction between the host and the pathogen in chronic infections is long-lasting where the host is unable to deal with the pathogen over time and the pathogen feeds from the energetic reserve from the host and/or use host cells to replicate^3,34^. These interactions between parasite-host slowly deteriorate the host health causing slow growth and poor performance, as proposed for other microsporidians causing growth retardation in the bee *Apis cerana*^32^. Particularly, microsporidians have been reported to cause chronic persistent diseases that can vary in their infection intensity throughout the disease cycle^32,33^, and in some cases, the pathogen cannot be detected in the infected organisms. However, infected organisms lacking clinical signs are able to transmit the infection^33^. This information strongly supports our findings related to the disease pattern of EHP in *P. vannamei* where infection intensity can significantly decrease at late stage of the disease. It is interesting to point out, that this cyclic disease patter may help explain why some studies fail to detect EHP in some shrimp displaying white feces syndrome^35^. This syndrome has been associated to EHP under particular conditions and coinfections with certain strains of *Vibrio parahaemolyticus*^36^.

Our data show that the cyclic pattern of EHP infection status is associated with a chronic infection that progressively deteriorates the microbiome community and its functionality, resulting in an outbreak of pathogenic and opportunistic bacteria and fungus. The phyla frequency of bacteria did not register a clear relation with the disease stage. However, in fungi, an increase of the Basidiomycota phyla accompanied by a decrease of the Ascomycota phyla was registered at the end of the development-stage and the late-stage of the disease. The Ascomycota phylum was registered at the early-stage of the disease, which was expected as it is the most abundant phyla in a healthy shrimp hepatopancreas^28^. The increase of the Basidiomycota phyla at the late-stage was associated with an increase of the genus *Malasseiza*, which has been associated as a pathogen of marine organisms^37^. This suggests that pathogenic fungi from Basidiomycota phyla proliferate in EHP-infected shrimp gradually replacing beneficial fungi from the Ascomycota phyla. It was interesting to note that the Microsporidian phylum was not registered in our curated fungal sequences originating from EHP-infected shrimp. This was attributed to the absence of EHP ITS amplification. Although, ITS is proposed as a universal DNA barcode biomarker for fungi^38^, microsporidian rDNA units are dispersed throughout the genome in some species, resulting in the non-amplification of the ITS region^39^.

At a bacterial genus level, a higher abundance of *Bacteroides*, *Pseudomonas*, *Acinetobacter*, *Delftia*, *Agrobacterium*, *Achromobacter* and *Commamonas* was recorded at the early-stage and clustered within them. The clustering between these bacteria indicates that they may be related to a similar environmental condition in a particular habitat (hepatopancreas) and to a healthy status of the host as all these taxa have been registered in healthy *P. vannamei* shrimp^11,40–42^. The beneficial activity of these taxa is highly related to their previously reported functions in the host digestive system. The genera *Bacteroides* and *Comamonas* spp. have been reported to be major producers of vitamin B12^43^ along with antimicrobial metabolites in *Comamonas* spp.^44,45^. The *Pseudomonas* genus has been related to increase in digestive enzymes activity^11^ and pathogen resistance^40^ in *P. vannamei*. The genera *Acrhomobacter* and *Acinetobacter* have been reported in shrimp gut and include nitrifying and denitrifying bacteria, which may be related to the improvement of water quality^46,47^. Bacteria from the genus *Agrobacterium* have been related to increased lipid absorption in alpine trout (*Salvelinus alpinus*)^48^. Finally, the genera *Delftia* have been used to reduce the virulence of gram-negative pathogens^49^. Additionally, the loss of members of the families Comamonadaceae and Bacteroides genera have been previously reported in *P. vannamei* infected with EHP^42^. The above supports the hypothesis that those bacterial taxa from the early-stage of the disease are essential for health and improvement of the digestive system homeostasis in *P. vannamei*. This also suggests that EHP modified the hepatopancreas environment which results in the displacement of these beneficial bacteria.

It is worth noting even though *Acinetobacter* and *Delfia* genera were registered in the early-stage and have been associated with healthy shrimp, other authors have reported that those genera can be pathogenic for shrimp^50,51^. This indicates a healthy microbiome balance not only depends on the absence or presence of certain taxa but also on their abundance^12^. Among fungi, only the genus *Komagataella* was associated with the early-stage of the disease related to the species *K. phaffii*. However, to our knowledge, it has not been reported in *P. vannamei* shrimp and is the first time that is being related to a good health status in *P. vannamei*.

Contrary to the early-stage, during the developmental stage, a lower number of bacterial taxa was differentially expressed compared to fungi suggesting a higher proliferation of fungi species at this stage. Low over-representation of bacteria taxa may be a consequence of antagonistic interactions among beneficial, commensal and pathogenic microorganisms as the disease begins to progress. Only one bacterial taxon was overrepresented at the development-stage of the disease and corresponded to class Actinobacteria. This class has been detected in healthy shrimp^11^, related to antifungal properties^52^, antagonistic activity against pathogenic *Vibrio* spp.^53^ and related to shrimp infected with EHP^42^. This suggests a higher modulation of the Actinobacteria class due to EHP proliferation during the development-stage of the disease. Considering the antifungal properties of this class^52^, its overrepresentation could be explained as it is harder to displace these bacteria by EHP or other opportunistic and pathogenic fungus genera such *Naganisha* and *Alternaria* which were the most overrepresented fungi taxa at the development-stage. The effect of *Naganishia* and *Alternaria* genera on the health of *P. vannamei* shrimp has not been clearly assessed yet. However, these fungi are considered opportunistic pathogens in humans^28,54^. This indicates that during the development-stage of the disease the shrimp’s digestive systems start to lose the community structure and balance of the microbiome. The development stage could be described as a transition zone, where beneficial bacteria is being displaced and opportunistic fungi begins to proliferate.

At the late-stage of the disease, a displacement of beneficial bacteria was very evident and proliferation of the pathogenic bacteria genera like *Vibrio* and *Aquimarina* and pathogenic fungus genera like *Malassezia* were registered. These bacterial and fungal genera have been considered opportunistic pathogens^37,55,56^ supporting the hypothesis that EHP at the late-stage of the disease generates a dysbiosis process in the hepatopancreas of *P. vannamei* that enables proliferation of opportunistic microorganisms. An increase of vibrio abundance have been related to dysbiosis^10^, and EHP infection in *P. vannamei*^27^. The genera *Aquimarina* has been also related to disease in crustaceans causing mortalities in *P. vannamei* larvae^57^ and increased abundance of *Malassezia* sp. has been reported in white feces disease in *P. vannamei*^58^ that can be caused due to coinfection with EHP and *V. parahaemolyticus*^36^. Further investigation will unravel role of *Aquimarina* and *Malassezia* in shrimp health and understanding if these bacteria are commensals that turn pathogenic once the EHP contributes to specific host environmental changes. Interestingly the bacteria genera *Leadbetterella* was also overrepresented at the late-stage. However, due to this genera being linked to healthy shrimp treated with probiotics^59^, its role in shrimp needs to be further investigated.

Based on our data, as exposure time with EHP progresses, beneficial microorganisms showed reduce abundance or are displaced and commensal, opportunistic and pathogen start to proliferate. Also, some microorganisms cannot be considered as beneficial or pathogenic as their role will depend on the physico-chemical properties of the digestive system^60^ and the interactions with the community of the microbiome^60^ which are being altered by EHP. It is important to note that while some microbiome changes could be related to SPF shrimp adjusting to new environmental conditions (tank in which the animals were reared) the microbiome community/functions that were observed strongly support that EHP is the main driver of hepatopancreas microbiome/microenvironment changes. Particularly, the proliferation of opportunistic/pathogenic microorganisms was more evident at the late stage in organisms with higher infection grades compared with those with lower infection grades. Furthermore, as mentioned above, the existing literature on microbiota alteration and the presence of certain microorganisms related to EHP infection in penaeids align with our observations.

The differences in microorganism abundances (higher differentially expressed taxa at the early-stage) registered between disease stages, were according to the results obtained from the diversity index in bacteria. Although no significant differences were found in the bacterial alpha index among disease stages, the beta index significantly separated the early-stage from the development and late-stage of the disease indicating a shift in the microbiome community and diversity^12^. As expected, the lower diversity bacteria values were registered at the late-stage of the disease. Similar findings have been previously reported where lower alpha diversity values are registered in diseased organisms, but no significant differences were detected^42^. This supports the fact that diseased condition reduces bacterial alpha diversity in *P. vannamei* digestive system which is accompanied by dysbiosis^10,12,61^. The reduction in microorganisms’ diversity is attributed to opportunistic pathogens that outcompete commensal and beneficial bacteria, and cause and outbreak in the shrimp digestive system^12,61^. It must be highlighted that in our study the microbiome diversity of SPF shrimp before the infection was the lowest recorded in all the experimental trials, which may increase variability in the experimental design when assessing microorganisms’ diversity. This low microbial diversity of the SPF shrimp was attributed to the fact that laboratory maintained animals are not exposed to a high diversity of microorganisms, unlike farmed shrimp^13^.

Contrary to bacteria, the alpha diversity in fungi increased at the late-stage of the disease. The reduction of diversity in fungi has been reported in diseased shrimp^28^. In our study, the increase in diversity was associated with a proliferation of opportunistic pathogenic fungi at the late-stage which was related to member of the Basidomicota phylum. The abundance of the members belonging to this phylum has been considered as an indicator of dysbiosis in shrimp^61^. The inverse pattern in fungal diversity compared to bacteria may be associated with a possible improvement of the hepatopancreas environment caused by EHP for opportunistic pathogenic fungi. It is known that microsporidian damages the hepatopancreas cells^3^ this may increase dead hepatopancreatic epithelial cells that sloughed off into the lumen of hepatopancreatic tubule. We speculate that the detachment of epithelial cells from the basal membrane exposes the underlying tissue and thus creating a right niche for the proliferation of the Basidomicota phyla.

The predicted functionality of the microbiome was closely related to the overrepresented taxa at each stage of EHP infection cycle. As mentioned earlier, the bacteria and fungi taxa at the early-stage of the disease were highly associated with nutrient acquisition which was supported by the enriched metabolic pathway results. At the early-stage of the disease, the overrepresented pathways in bacteria and fungi were ABC transporters and fatty acid biosynthesis which are highly associated with digestion and absorption of nutrients but also to higher performance of the defense systems^41,62^. Some of these microbiome functions have been reported in healthy shrimp^40^ supporting the importance of microorganisms in nutrient acquisition and the health of the host^41^.

At the development-stage, which could be considered as a transition zone, a healthy microbiome starts to lose balance due to an increase in interactions between beneficial and opportunistic pathogenic microorganisms. However, at the late-stage of the disease, a loss of metabolic pathways related to nutrient acquisition was registered for members of both bacteria and fungi domains. This was previously postulated by Duan et al.^42^ who mention that EHP may influence nutrient absorption by intestine bacteria that corroborates the effect of EHP infection over the nutrient intake by shrimp. Interestingly, at the late-stage of the disease, the enriched metabolic pathways from bacteria and fungi were completely different. In bacteria, metabolic pathways were related to pathogenic bacteria proliferation (*Vibrio cholerae* pathogenic cycle, peptidoglycan biosynthesis, homologous recombination). The reported immunosuppression in *P. vannamei* infected by EHP, which is accompanied by a dysbiosis process^27^, could explain the proliferation of pathogenic bacteria (*Vibrio* and *Aquimarina* genera) that were registered in this study.

**Figure 8:**
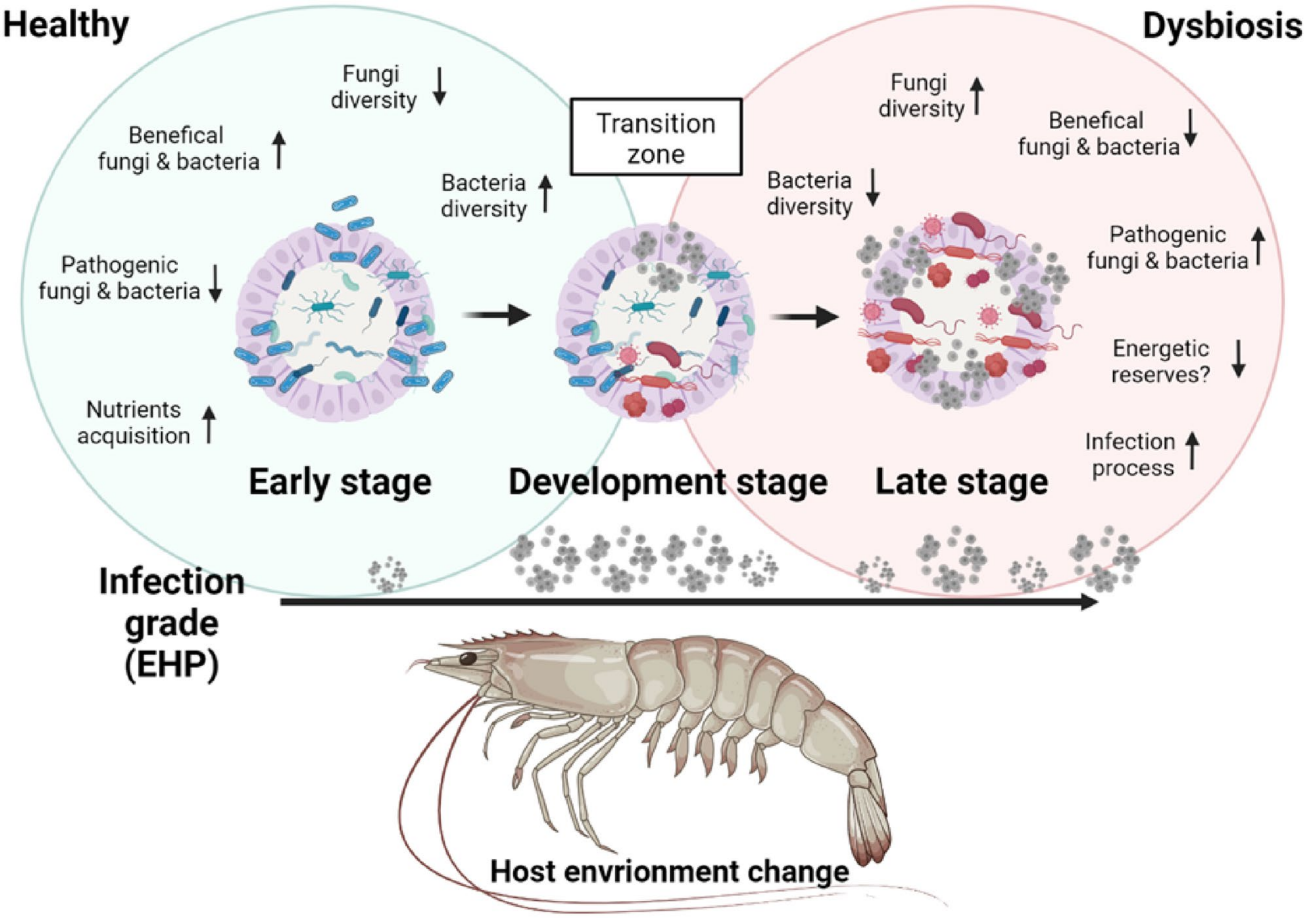
Modulation of the microbiome in the hepatopancreas of shrimp (*P. vannamei*) infected with EHP. Schematic diagram that represents the main changes produced in the host hepatopancreatic environment during the infection of EHP (30 days) according to the disease stages registered in this study. Infection intensity represents the cyclic patter of EHP, where bigger EHP figures represent higher infection grade and smallest EHP figures represent lower infection grade. Blue bacteria: beneficial microbiota; Gray dots: EHP; Red bacteria: opportunistic and pathogenic microbiota. This image was created with BioRender (http://biorender.com/).

In fungi, most of the enriched metabolic pathways were associated with carbohydrates metabolisms including glycan degradation. This suggests that fungus at the late-stage may take advantage of the carbohydrate reserves of EHP-infected shrimp. This hypothesis is supported by transcriptomic, proteomic and metabolomic analyses where energy metabolism pathway such as carbohydrates degradation, digestion and absorption are regulated by EHP infection^42,63^. Based on our findings, we propose EHP infection results in a cyclic chronic degenerative process that causes dysbiosis (Fig. 8). We speculate that EHP proliferates when the hepatopancreas reserves are high achieving a high infection intensity (I4) and as nutrient reserves are depleted pathogen proliferation halts or decreases. If conditions are favorable shrimp will temporarily recover and the hepatopancreas will once again become favorable niche for EHP.

## Conclusions

Hepatopancreatic microsporidiosis caused by EHP in *P. vannamei* can be cataloged as a cyclic chronic degenerative infection where the healthy microbiome begins to lose diversity, beneficial microorganisms, and functionality related to nutrient acquisition and host health (dysbiosis). The longer EHP infection continues at a higher infection intensity a more evident alteration of the hepatopancreas microbiome will be observed. We determined EHP infection favors the increase in the abundance of bacterial from genera *Vibrio*, *Aquimarina* and *Leadbetterella* and in the fungal genus, *Malassezia*. Candidate beneficial microorganisms that are displaced during EHP infection include beneficial bacteria such as *Bacteroides*, *Pseudomonas, Acinetobacter*, *Delftia*, *Agrobacterium*, *Achromobacter, Comamonas* as well as species belonging to the family *Bacillaceae*, and the fungal genus *Komagataella*. Some members of these taxa’s could be cultured and employed as probiotics to alleviate HPM disease progression. In addition, this is the first time, a quantitative histological approach was used to study the EHP infection cycle in the context of the microbiome changes in the shrimp hepatopancreas.

## Data Availability

16S rRNA V3-V4 and ITS amplicon sequencing datasets that support the conclusions of this article were deposited in NCBI under accession number PRJNA823752. All other relevant data are included as supporting information files and within the manuscript.
